# Supplementary material for: Neuropsychological Effects of the Lockdown Due to the COVID-19 Pandemic on Patients with Alzheimer’s Disease and Their Caregivers: The “ACQUA” (Alzheimer–COVID QUArantine Questionnaire) Study
Source: Int J Environ Res Public Health. 2024 Dec 4;21(12):1622. doi: 10.3390/ijerph21121622 (PMC11675157; doi:10.3390/ijerph21121622)
Supplement: Supplementary file 1 [file ijerph-21-01622-s001.zip › ijerph-3228968-supplementary.pdf]

"ACQUA" (Alzheimer-Covid QUArantine questionnaire)

PART 1
DEMOGRAPHIC DATA and HEALTH STATUS (1-27)

CAREGIVER (___________________)

1. Caregiver's sex: M/F
2. How old are you? ____
3. How many years of schooling have you completed? ____
   a. PRIMARY SCHOOL
   b. SECONDARY SCHOOL
   c. UNIVERSITY/DOCTORATE/MASTER'S
4. What is your marital status?
   a. MARRIED/LIVING TOGETHER
   b. SEPARATED/WIDOWED
   c. SINGLE
5. How many children do you have? ____
6. Do you work? YES/NO
7. If YES, what is your job? ____
8. What is your relationship to the patient?
   a. WIFE/HUSBAND
   b. SON/DAUGHTER
   c. BROTHER/SISTER
   d. OTHER FAMILY RELATION
   e. NO FAMILY RELATION

PATIENT (___________________)

9. Patient's sex: M/F
10. How old is the patient? ____
11. How many years of schooling has the patient completed? ____
    a. PRIMARY SCHOOL
    b. SECONDARY SCHOOL
    c. UNIVERSITY/DOCTORATE/MASTER'S
12. What is the patient's marital status?
    a. MARRIED/LIVING TOGETHER
    b. SEPARATED/WIDOWED
    c. SINGLE
13. How many children does the patient have? ____
14. Has the patient ever worked? YES/NO
15. If YES, what was their main job? ____
16. What is the patient's current health status regarding the coronavirus disease?
    a. I AM CURRENTLY SICK
    b. I HAVE NEVER CONTRACTED THE VIRUS
    c. PREVIOUS INFECTION, FULL RECOVERY
    d. PREVIOUS INFECTION, LONG-TERM EFFECTS
    (___________________________________)
17. Has the patient ever had a COVID-19 test? YES/NO
18. How many tests did the patient take during the lockdown? _____
19. How many of these tests were positive? _____

20. Has the patient ever been placed in quarantine during this period? YES/NO
21. What is the current health condition of the patient regarding the coronavirus disease?
    a. THE PATIENT IS CURRENTLY SICK
    b. THE PATIENT HAS NEVER CONTRACTED THE VIRUS
    c. PREVIOUS INFECTION, FULL RECOVERY
    d. PREVIOUS INFECTION, LONG-TERM EFFECTS (________________________________)
22. Has the patient ever had a COVID-19 test? YES/NO
23. How many tests has the patient taken during the lockdown? _____
24. How many of these tests were positive? _____
25. Has the patient ever been placed in quarantine during this period? YES/NO
26. Since the introduction of containment measures, has there been a need to contact:
    a. the family doctor
    b. the referring doctor of the CDCD
    c. both
    d. neither
27. As a result of contact with the family doctor and/or the CDCD doctor, was the treatment modified?
    a. Yes, with benefit
    b. Yes, without benefit
    c. No change in treatment

**Notes*

____
____
____
____
____
____
____
____
____
____
____
____
____
____
____

PART 2
CHANGES IN THE MANAGEMENT OF CARE (28-48)

28. Did you live in with the patient before the containment measures were enforced? YES/NO
29. How many times a week did you see the patient before the containment measures were enforced?
    a. DAILY
    b. AT LEAST 3 TIMES A WEEK
    c. 1-2 TIMES A WEEK
    d. LESS THAN ONCE A WEEK
30. Has the frequency of your visits changed since the containment measures were enforced? YES/NO
    If YES
    a. It has increased A LOT
    b. It has decreased A LOT
    c. It has increased A LITTLE
    d. It has decreased A LITTLE
31. Before the pandemic and the implementation of the containment measures, did you have phone contact with the patient? YES/NO
32. How often did these phone contacts occur?
    a. MULTIPLE TIMES A DAY
    b. DAILY
    c. AT LEAST 3 TIMES A WEEK
    d. 1-2 TIMES A WEEK
    e. LESS THAN ONCE A WEEK
33. Has the frequency of your phone contacts with the patient changed since the containment measures were enforced? YES/NO
    If YES
    a. It has increased A LOT
    b. It has decreased A LOT
    c. It has increased A LITTLE
    d. It has decreased A LITTLE
34. Did you continue working after the containment measures were enforced? YES/NO
    If YES
    a. My on-site working hours remained the same
    b. My on-site working hours decreased
    c. I no longer work on-site, I work remotely (SMART WORKING)
    If NO
    d. I did not work before the lockdown
    e. I lost my job due to the pandemic
35. How many people do you live with? ____
36. Does your home have a separate habitable room for each person? YES/NO
37. Has your economic situation changed in a way that you consider concerning since the containment measures were enforced? YES/NO
38. How many people does the patient live with? ____
39. Does the patient's home have a separate habitable room for each person? YES/NO
40. Has the patient's economic situation changed in a way that you consider concerning since the containment measures were enforced? YES/NO
41. Did the patient have professional care support (e.g., caregiver) before the epidemic? YES/NO

42. If YES, has the need for care changed since the containment measures were enforced? YES/NO
    If YES, how much has it changed?
    a. It has increased A LOT
    b. It has decreased A LOT
    c. It has increased A LITTLE
    d. It has decreased A LITTLE
43. Has the frequency of the patient's outings changed since the containment measures were enforced? YES/NO
    If YES,
    a. It has increased A LOT
    b. It has decreased A LOT
    c. It has increased A LITTLE
    d. It has decreased A LITTLE
    e. SUSPENDED
44. Before the containment measures were enforced, was the patient undergoing rehabilitation therapy? YES/NO
45. If YES, has the frequency of the rehabilitation therapy changed since the containment measures were enforced? YES/NO
    If YES, how much has it changed?
    a. It has increased A LOT
    b. It has decreased A LOT
    c. It has increased A LITTLE
    d. It has decreased A LITTLE
    e. SUSPENDED
46. Before the containment measures were enforced, did the patient engage in cognitively stimulating social activities? YES/NO
    If YES, what were they?
    a. ____
    b. ____
    c. ____
    d. ____
47. If YES, has the frequency of the patient's cognitively stimulating social activities changed since the containment measures were enforced? YES/NO
    If YES, how much has it changed?
    a. It has increased A LOT
    b. It has decreased A LOT
    c. It has increased A LITTLE
    d. It has decreased A LITTLE
    e. SUSPENDED
48. Has the frequency of the patient's outings changed since the containment measures were enforced? YES/NO
    If YES,
    a. It has increased A LOT
    b. It has decreased A LOT
    c. It has increased A LITTLE
    d. It has decreased A LITTLE
    e. SUSPENDED

***Notes**

____
____
____
____
____
____
____
____
____
____
____
____
____
____
____

PART 3
EFFECTS OF THE LOCKDOWN ON THE PATIENT (49-127)

49. Have the patient's neurological clinical conditions changed compared to the 4 weeks prior to the enforcement of the containment measures (LOCKDOWN)? YES/NO
    If YES, how have they changed?
    a. Improved A LOT
    b. Worsened A LOT
    c. Improved A LITTLE
    d. Worsened A LITTLE
50. Compared to before the enforcement of the containment measures, has the patient developed new cognitive symptoms? YES/NO
    If YES, which ones?
    a. Memory
    b. Attention
    c. Language
    d. Executive functions
    e. Spatial orientation
    f. Temporal orientation
    g. Topographical orientation
51. [**Memory**]: Was the symptom present before the lockdown? YES/NO
    If YES,
    a. It has worsened
    b. It has improved
    c. It has remained unchanged
52. [**Memory**] Rate the severity of this symptom:
    a. VERY MILD
    b. MILD
    c. MODERATE
    d. SEVERE
53. [**Memory**] How concerned about this symptom?
    a. Not at all
    b. A little
    c. Somewhat
    d. Very
    e. Extremely
54. [**Language**]: Was the symptom present before the lockdown? YES/NO
    If YES,
    a. It has worsened
    b. It has improved
    c. It has remained unchanged
55. [**Language**] Rate the severity of this symptom:
    a. VERY MILD
    b. MILD
    c. MODERATE
    d. SEVERE
56. [**Language**] How concerned about this symptom?
    a. Not at all
    b. A little
    c. Somewhat

d. Very
e. Extremely

57. [**Executive functions**]: Was the symptom present before the lockdown? YES/NO
If YES,
a. It has worsened
b. It has improved
c. It has remained unchanged
58. [**Executive functions**] Rate the severity of this symptom:
a. VERY MILD
b. MILD
c. MODERATE
d. SEVERE
59. [**Executive functions**] How concerned about this symptom?
a. Not at all
b. A little
c. Somewhat
d. Very
e. Extremely
60. [**Spatial orientation**]: Was the symptom present before the lockdown? YES/NO
If YES,
a. It has worsened
b. It has improved
c. It has remained unchanged
61. [**Spatial orientation**] Rate the severity of this symptom:
a. VERY MILD
b. MILD
c. MODERATE
d. SEVERE
62. [**Spatial orientation**] How concerned about this symptom?
a. Not at all
b. A little
c. Somewhat
d. Very
e. Extremely
63. [**Temporal orientation**]: Was the symptom present before the lockdown? YES/NO
If YES,
a. It has worsened
b. It has improved
c. It has remained unchanged
64. [**Temporal orientation**] Rate the severity of this symptom:
a. VERY MILD
b. MILD
c. MODERATE
d. SEVERE
65. [**Temporal orientation**] How concerned about this symptom?
a. Not at all
b. A little
c. Somewhat
d. Very
e. Extremely

66. [**Topographical orientation**]: Was the symptom present before the lockdown? YES/NO
    If YES,
    a. It has worsened
    b. It has improved
    c. It has remained unchanged
67. [**Topographical orientation**] Rate the severity of this symptom:
    a. VERY MILD
    b. MILD
    c. MODERATE
    d. SEVERE
68. [**Topographical orientation**] How concerned about this symptom?
    a. Not at all
    b. A little
    c. Somewhat
    d. Very
    e. Extremely
69. Regarding the patient's behaviour, have you noticed the development of new symptoms since the containment measures were enforced? YES/NO
    If YES, which ones?
    a. Apathy
    b. Anxiety
    c. Depression
    d. Sleep disturbances
    e. Delusions
    f. Hallucinations
    g. Wandering
    h. Agitation
    i. Appetite changes
    j. Sundowning
    k. Changes in sexual behaviour/disinhibition
70. [**Apathy**]: Was the symptom present before the lockdown? YES/NO
    If YES,
    a. It has worsened
    b. It has improved
    c. It has remained unchanged
71. [**Apathy**] Rate the severity of this symptom:
    a. VERY MILD
    b. MILD
    c. MODERATE
    d. SEVERE
72. [**Apathy**] How concerned about this symptom?
    a. Not at all
    b. A little
    c. Somewhat
    d. Very
    e. Extremely
73. [**Anxiety**]: Was the symptom present before the lockdown? YES/NO
    If YES,
    a. It has worsened

b. It has improved
c. It has remained unchanged

74. [**Anxiety**] Rate the severity of this symptom:
a. VERY MILD
b. MILD
c. MODERATE
d. SEVERE

75. [**Anxiety**] How concerned about this symptom?
a. Not at all
b. A little
c. Somewhat
d. Very
e. Extremely

76. [**Depression**]: Was the symptom present before the lockdown? YES/NO
If YES,
a. It has worsened
b. It has improved
c. It has remained unchanged

77. [**Depression**] Rate the severity of this symptom:
a. VERY MILD
b. MILD
c. MODERATE
d. SEVERE

78. [**Depression**] How concerned about this symptom?
a. Not at all
b. A little
c. Somewhat
d. Very
e. Extremely

79. [**Sleep disturbances**]: Was the symptom present before the lockdown? YES/NO
If YES,
a. It has worsened
b. It has improved
c. It has remained unchanged

80. [**Sleep disturbances**] Rate the severity of this symptom:
a. VERY MILD
b. MILD
c. MODERATE
d. SEVERE

81. [**Sleep disturbances**] How concerned about this symptom?
a. Not at all
b. A little
c. Somewhat
d. Very
e. Extremely

82. [**Delusions**]: Was the symptom present before the lockdown? YES/NO
If YES,
a. It has worsened
b. It has improved
c. It has remained unchanged

83. [**Delusions**] Rate the severity of this symptom:
    a. VERY MILD
    b. MILD
    c. MODERATE
    d. SEVERE
84. [**Delusions**] How concerned about this symptom?
    a. Not at all
    b. A little
    c. Somewhat
    d. Very
    e. Extremely
85. [**Hallucinations**]: Was the symptom present before the lockdown? YES/NO
    If YES,
    a. It has worsened
    b. It has improved
    c. It has remained unchanged
86. [**Hallucinations**] Rate the severity of this symptom:
    a. VERY MILD
    b. MILD
    c. MODERATE
    d. SEVERE
87. [**Hallucinations**] How concerned about this symptom?
    a. Not at all
    b. A little
    c. Somewhat
    d. Very
    e. Extremely
88. [**Agitation**]: Was the symptom present before the lockdown? YES/NO
    If YES,
    a. It has worsened
    b. It has improved
    c. It has remained unchanged
89. [**Agitation**] Rate the severity of this symptom:
    a. VERY MILD
    b. MILD
    c. MODERATE
    d. SEVERE
90. [**Agitation**] How concerned about this symptom?
    a. Not at all
    b. A little
    c. Somewhat
    d. Very
    e. Extremely
91. [**Wandering**]: Was this symptom already present before the lockdown? YES/NO
    If YES,
    a. It has worsened
    b. It has improved
    c. It has not changed
92. [**Wandering**]: How would you rate the severity of this symptom?
    a. VERY MILD
    b. MILD

c. MODERATE
d. SEVERE

93. [**Wandering**]: How concerned about this symptom?
a. Not at all
b. A little
c. Fairly
d. Very
e. Extremely

94. [**Appetite**]: Was this symptom already present before the lockdown? YES/NO
If YES,
a. It has worsened
b. It has improved
c. It has not changed

95. [**Appetite**]: How would you rate the severity of this symptom?
a. VERY MILD
b. MILD
c. MODERATE
d. SEVERE

96. [**Appetite**]: How concerned about this symptom?
a. Not at all
b. A little
c. Fairly
d. Very
e. Extremely

97. [**Sundowning**]: Was this symptom already present before the lockdown? YES/NO
If YES,
a. It has worsened
b. It has improved
c. It has not changed

98. [**Sundowning**]: How would you rate the severity of this symptom?
a. VERY MILD
b. MILD
c. MODERATE
d. SEVERE

99. [**Sundowning**]: How concerned about this symptom?
a. Not at all
b. A little
c. Fairly
d. Very
e. Extremely

100. [**Disinhibition**]: Was this symptom already present before the lockdown? YES/NO
If YES,
a. It has worsened
b. It has improved
c. It has not changed

101. [**Disinhibition**]: How would you rate the severity of this symptom?
a. VERY MILD
b. MILD
c. MODERATE
d. SEVERE

102. [**Disinhibition**]: How concerned about this symptom?
    a. Not at all
    b. A little
    c. Fairly
    d. Very
    e. Extremely
103. Compared to before the lockdown measures, do you think there have been changes in patient's ability to use common communication tools (landline phone, cell phone, etc.)? YES/NO
    If YES, how much?
    a. It has increased A LOT
    b. It has decreased A LOT
    c. It has increased A LITTLE
    d. It has decreased A LITTLE
104. AUTONOMY: YES/NO
105. Compared to before the lockdown measures, do you think there have been changes in patient's ability to manage the household and organize and serve properly prepared meals? YES/NO
    If YES, how much?
    a. It has increased A LOT
    b. It has decreased A LOT
    c. It has increased A LITTLE
    d. It has decreased A LITTLE
106. AUTONOMY: YES/NO
107. Compared to before the lockdown measures, do you think there have been changes in patient's ability to remember and take medication regularly? YES/NO
    If YES, how much?
    a. It has increased A LOT
    b. It has decreased A LOT
    c. It has increased A LITTLE
    d. It has decreased A LITTLE
108. AUTONOMY: YES/NO
109. Compared to before the lockdown measures, have you noticed changes in patient's ability to keep track of household finances? YES/NO
    If YES, how much?
    a. It has increased A LOT
    b. It has decreased A LOT
    c. It has increased A LITTLE
    d. It has decreased A LITTLE
110. AUTONOMY: YES/NO
111. Compared to before the lockdown measures, do you think there have been changes in patient's ability to make an adequate shopping list and plan shopping correctly? YES/NO
    If YES, how much?
    a. It has increased A LOT
    b. It has decreased A LOT
    c. It has increased A LITTLE
    d. It has decreased A LITTLE
112. AUTONOMY: YES/NO
113. Compared to before the lockdown measures, do you think there have been changes in patient's ability to use household appliances independently? YES/NO

If YES, how much?
a. It has increased A LOT
b. It has decreased A LOT
c. It has increased A LITTLE
d. It has decreased A LITTLE

114. AUTONOMY: YES/NO
115. Compared to before the lockdown measures, do you think there have been changes in patient's ability to maintain hobbies? YES/NO
If YES, how much?
a. It has increased A LOT
b. It has decreased A LOT
c. It has increased A LITTLE
d. It has decreased A LITTLE
116. AUTONOMY: YES/NO
117. Compared to before the lockdown measures, do you think there have been changes in patient's ability to maintain personal hygiene? YES/NO
If YES, how much?
a. It has increased A LOT
b. It has decreased A LOT
c. It has increased A LITTLE
d. It has decreased A LITTLE
118. AUTONOMY: YES/NO
119. Compared to before the lockdown measures, do you think there have been changes in patient's ability to dress him/herself? YES/NO
If YES, how much?
a. It has increased A LOT
b. It has decreased A LOT
c. It has increased A LITTLE
d. It has decreased A LITTLE
120. AUTONOMY: YES/NO
121. Compared to before the lockdown measures, do you think there have been changes in patient's ability to control bowel/incontinence? YES/NO
If YES, how much?
a. It has increased A LOT
b. It has decreased A LOT
c. It has increased A LITTLE
d. It has decreased A LITTLE
122. AUTONOMY: YES/NO
123. Compared to before the lockdown measures, do you think there have been changes in patient's ability to move around the house and outside? YES/NO
If YES, how much?
a. It has increased A LOT
b. It has decreased A LOT
c. It has increased A LITTLE
d. It has decreased A LITTLE
124. AUTONOMY: YES/NO
125. Compared to before the lockdown measures, do you think there have been changes in patient's ability to wash clothes and to change them everyday? YES/NO
If YES, how much?
a. It has increased A LOT
b. It has decreased A LOT

c. It has increased A LITTLE
d. It has decreased A LITTLE

126. AUTONOMY: YES/NO
127. Regarding awareness of the pandemic and the ongoing restrictions:
    a. The patient spontaneously remembers that the COVID-19 health emergency is ongoing
    b. Remembers which activities they cannot do or must do differently due to the emergency
    c. Maintains appropriate behaviour regarding restrictions
    d. Spontaneously talks about quarantine
    e. Spontaneously engages in appropriate behaviours even if they do not explicitly remember that the emergency is ongoing

**Notes*

PART 4
EFFECTS OF THE LOCKDOWN ON CAREGIVERS (128-157)

128. Thinking about yourself before the outbreak of the epidemic and before starting your role as a caregiver, would you say you are generally an optimistic person? YES/NO
129. Thinking about yourself before the outbreak of the epidemic and before starting your role as a caregiver, did you normally wake up in the morning with a positive desire to start your day? YES/NO
130. Thinking about yourself before the outbreak of the epidemic and before starting your role as a caregiver, was your mood generally quite good? YES/NO
131. Do you consider yourself a person who is interested in analysing and understanding your emotions? YES/NO
132. Thinking about before the epidemic, do you feel that your caregiving duties negatively impacted your life? YES/NO
133. Before the epidemic, did you feel that your caregiving tasks confronted you with problems that you feared you wouldn't be able to handle? YES/NO
134. Are you afraid of the coronavirus? (of getting infected, becoming seriously ill, infecting others, etc.) YES/NO If YES, how much?
    a. VERY LITTLE
    b. LITTLE
    c. MODERATELY
    d. A LOT
135. With the introduction of measures to contain the spread of the virus, you found yourself dealing with unpleasant emotions that are difficult to bear.
    a. Strongly agree
    b. Agree
    c. Neutral
    d. Disagree
    e. Strongly disagree
136. Compared to before, you missed companionship.
    a. Strongly agree
    b. Agree
    c. Neutral
    d. Disagree
    e. Strongly disagree
137. Compared to before, you missed time for yourself.
    a. Strongly agree
    b. Agree
    c. Neutral
    d. Disagree
    e. Strongly disagree
138. Compared to before, you felt alone when solving problems.
    a. Strongly agree
    b. Agree
    c. Neutral
    d. Disagree
    e. Strongly disagree
139. Compared to before, you missed the emotional closeness of others.
    a. Strongly agree
    b. Agree
    c. Neutral

d. Disagree
e. Strongly disagree

140. With the introduction of measures to contain the virus, you would say that your daily life has worsened.
a. Strongly agree
b. Agree
c. Neutral
d. Disagree
e. Strongly disagree

141. Compared to before, which of the following symptoms or moods do you feel have appeared during the lockdown:
a. **Sadness** YES/NO
If YES, has it worsened?
i. NO
ii. Very little
iii. Little
iv. Moderately
v. A lot

b. **Feeling like crying** YES/NO
If YES, has it worsened?
i. NO
ii. Very little
iii. Little
iv. Moderately
v. A lot

c**. Lack of motivation** YES/NO
If YES, has it worsened?
i. NO
ii. Very little
iii. Little
iv. Moderately
v. A lot

d. **Irritability and nervousness** YES/NO
If YES, has it worsened?
i. NO
ii. Very little
iii. Little
iv. Moderately
v. A lot

e. **Feeling of hopelessness** YES/NO
If YES, has it worsened?
i. NO
ii. Very little
iii. Little
iv. Moderately
v. A lot

f. **Guilt** YES/NO
If YES, has it worsened?
i. NO
ii. Very little
iii. Little
iv. Moderately
v. A lot

g. **Low self-esteem** YES/NO
If YES, has it worsened?
i. NO
ii. Very little
iii. Little
iv. Moderately
v. A lot

142. Compared to before, in your emotional experience:
a. Do you experience unpleasant emotions?
i. As before
ii. More than before
iii. Less than before

b. Do you feel others are more distant?
i. As before
ii. More than before
iii. Less than before

c. Do you feel the absence of concrete help?
i. As before
ii. More than before
iii. Less than before

d. Do you feel alone when facing problems?
i. As before
ii. More than before
iii. Less than before

e. Do you have moments when you can think only of yourself?
i. As before
ii. More than before
iii. Less than before

f. Do you engage in activities dedicated to your well-being?
i. As before
ii. More than before
iii. Less than before

143. Compared to before, you managed to dedicate yourself to pleasant or useful activities for your well-being.
a. Strongly agree
b. Agree

   c. Neutral
   d. Disagree
   e. Strongly disagree

144. You consider the current situation to be, in some way, a positive opportunity.
   a. Strongly agree
   b. Agree
   c. Neutral
   d. Disagree
   e. Strongly disagree

145. You believe that, in some way, the quality of your life has improved.
   a. Strongly agree
   b. Agree
   c. Neutral
   d. Disagree
   e. Strongly disagree

146. Compared to before, you find your caregiving duties more burdensome.
   a. Strongly agree
   b. Agree
   c. Neutral
   d. Disagree
   e. Strongly disagree

147. Compared to before, you think the weight of caregiving has worsened.
   a. Strongly agree
   b. Agree
   c. Neutral
   d. Disagree
   e. Strongly disagree

148. Compared to before, caregiving seems more burdensome.
   a. Strongly agree
   b. Agree
   c. Neutral
   d. Disagree
   e. Strongly disagree

149. Compared to before, you feel capable of solving the problems you encounter in your caregiving role.
   a. Strongly agree
   b. Agree
   c. Neutral
   d. Disagree
   e. Strongly disagree

150. Compared to before, you feel the support of others in your caregiving tasks.
   a. Strongly agree
   b. Agree
   c. Neutral
   d. Disagree
   e. Strongly disagree

151. The new situation has been an opportunity to better solve old problems in managing the dementia.
   a. Strongly agree
   b. Agree
   c. Neutral

d. Disagree
e. Strongly disagree

152. The new situation has revealed new, unexpected competencies of the patient.
a. Strongly agree
b. Agree
c. Neutral
d. Disagree
e. Strongly disagree

153. The new situation has caused you to experience new negative feelings towards the patient.
a. Strongly agree
b. Agree
c. Neutral
d. Disagree
e. Strongly disagree

154. The new situation has caused you to experience new positive feelings towards the patient. a. Strongly agree
b. Agree
c. Neutral
d. Disagree
e. Strongly disagree

155. Compared to before, regarding the new feelings you've experienced towards the patient:
a. You haven't experienced any new feelings
b. You've experienced positive feelings, but only occasionally
c. You've experienced positive feelings frequently
d. You've experienced positive feelings very frequently
e. You've experienced positive feelings daily
f. You've experienced negative feelings, but only occasionally
g. You've experienced negative feelings frequently
h. You've experienced negative feelings very frequently
i. You've experienced negative feelings daily

156. Overall, you consider the quality of your relationship with the patient to have changed.
a. Strongly agree
b. Agree
c. Neutral
d. Disagree
e. Strongly disagree

157. Compared to before, has your quality of life worsened? YES/NO
If YES, by how much?
a. Very little
b. Little
c. Moderately
d. A lot

**Notes*

____

____
____
____
____
____
____
____
____
____
____
____
____

Date ________________

Interviewer ___________________________

*****To use footnotes to add considerations from the interviewees that may be considered useful or as an explanation/deepening of the answers given.*
